# Retraction: Integrating mechanisms of response and resistance against the tubulin binding agent Eribulin in preclinical models of osteosarcoma

**DOI:** 10.18632/oncotarget.28663

**Published:** 2024-10-11

**Authors:** Valerie B. Sampson, Nancy S. Vetter, Wendong Zhang, Pratima U. Patil, Robert W. Mason, Erika George, Richard Gorlick, Edward A. Kolb

**Affiliations:** ^1^Cancer Therapeutics Laboratory, Nemours Center for Cancer and Blood Disorders, Nemours/A.I. duPont Hospital for Children, Wilmington, DE, USA; ^2^Department of Pediatrics - Hematology and Oncology, The Children’s Hospital at Montefiore, The Albert Einstein College of Medicine, Bronx, NY, USA; ^3^Nemours Center for Childhood Cancer Research, Alfred I. duPont Hospital for Children, Wilmington, DE, USA; ^4^Department of Biological Sciences, University of Delaware, Newark, DE, USA

**This article has been retracted:** The first author, Valerie B. Sampson, has confirmed there was incorrect reporting of data for some of the figures of this publication:

I. Figure 1D and 1E, No Template Controls were included for all PCR experiments.

II. Figure 2B: Flow cytometry data needs validation.

III. Table 1: Flow cytometry data needs validation.

IV. Figure 3B, data for graph needs validation.

V. Figure 3D, data for graph needs validation.

VI. Figure 4B, No Template Controls were included for all PCR experiments.

VII. Figure 5C, No Template Controls were included for all PCR experiments.

She made the following statement: “I take sole responsibility for the errors and the incorrect reporting of data. These actions were unknown to all co-authors of this study. I apologize to the members of staff of Oncotarget, the reviewers who provided the reviews for the manuscripts, all who were involved in the publication of this manuscript and anyone who is impacted by my actions.”

Her affiliated institution, Nemours Children’s Hospital, has conducted an investigation. Given the nature of the irregularities that she has disclosed, their team of investigators believe that this article needs to be retracted. Oncotarget has attempted to contact all the authors of this paper, including Dr. Sampson, without success. Therefore, in agreement with the formal inquiry of Nemours Children’s Hospital, the Oncotarget Editors retracted this article.

Original article: Oncotarget. 2016; 7:86594–86607. 86594-86607. https://doi.org/10.18632/oncotarget.13358

